# One in Twenty: Prevalence and Outcomes of Patients Brought to EDs Under Québec's P-38 Mental Health Act—A Retrospective Cohort Study: Un patient sur vingt : prévalence et résultats chez les patients ayant été admis au service des urgences en vertu de la loi p-38 du Québec sur la santé mentale—Étude de cohorte rétrospective

**DOI:** 10.1177/07067437261468893

**Published:** 2026-07-27

**Authors:** Marie-Soleil Dumont, Valérie Boucher, Pier-Alexandre Tardif, Ann-Pier Gagnon, Axel Benhamed, Pierre-Gilles Blanchard, Audrey-Anne Dumais-Michaud, Marcel Émond, Eric Mercier

**Affiliations:** 1 Faculté des sciences sociales, 4440 Université Laval, Québec, Canada; 2 Faculté de médecine, 12369 Université Laval, Québec, Canada; 3 36896 Axe Santé des populations et pratiques optimales en santé, CHU de Québec-Université Laval Research Center, Québec, Canada; 4 École de travail social et de criminologie, Faculté des sciences sociales, 204255 Université Laval, Québec, Canada; 5 607356 VITAM—Centre de recherche en santé durable, Québec, Canada

**Keywords:** involuntary commitment, crisis intervention, mental disorders, mental health, mental health act

## Abstract

**Introduction:**

In Quebec, the P-38.001 Act (P-38) allows police officers or crisis workers, under exceptional circumstances, to bring individuals at risk of harming themselves or others to the Emergency Department (ED) against their will. Data on the application of this Act remains limited.

**Objectives:**

We aimed to determine the prevalence of P-38 use among ED patients with mental health presentations and to compare their characteristics, clinical course, and outcomes with those who sought care voluntarily.

**Methods:**

We conducted a retrospective cohort study using a Public Health Agency of Canada–approved mental health database. Patients aged ≥14 years presenting to 5 EDs for mental health reasons in 2020–2021 were included. The main outcome, enforcement of P-38, was abstracted from medical charts. Information on care trajectory and management was also collected. Relative risks (RR) were estimated using modified Poisson regression with robust variance.

**Results:**

Among 15,021 mental health–related ED visits, 860 (5.7% [95% CI: 5.4-6.1]) involved enforcement of the P-38 Act. Mean age was 41.1 ± 16.5 years, with 46.1% aged 25-44, and 54.4% being male. Most lived independently (85.4%), arrived by ambulance (80.5%), and had a documented mental health history (92.2%). A higher proportion were materially deprived (*P* = 0.020). Compared with patients who voluntarily consulted in the ED, those under P-38 were more likely to be restrained (RR: 1.68 [95% CI: 1.45-1.94]), placed under preventive confinement (RR 3.41 [95% CI: 2.96-3.93]), receive multidisciplinary assessment (RR: 1.70 [95% CI: 1.59-1.80]), and be hospitalized (RR: 1.36 [95% CI: 1.24-1.48]).

**Conclusion:**

Enforcement of P-38 accounted for nearly 6% of mental health–related ED visits, disproportionately affecting materially deprived individuals in early to mid-adulthood with prior mental health disorders. Our findings suggest a reliance on coercive and short-term measures, underscoring the need for targeted policy efforts to expand community-based crisis intervention alternatives for this vulnerable population.

## Résumé

**Introduction:**

Au Québec, la loi P-38.001 (P-38) permet aux agents de la paix ou aux intervenants d’un service d’aide en situation de crise, dans des circonstances exceptionnelles, d’envoyer au service des urgences, contre leur gré, les personnes qui présentent un danger pour elles-mêmes ou pour autrui. Les données sur l’application de cette loi demeurent limitées.

**Objectifs:**

Nous avons cherché à déterminer la fréquence du recours à la loi P-38 pour amener des patients qui présentent un problème de santé mentale au service des urgences et à comparer leurs caractéristiques, leur évolution clinique et leurs résultats avec ceux des patients qui ont sollicité des soins de façon volontaire.

**Méthodes:**

Nous avons mené une étude de cohorte rétrospective à l’aide d’une base de données sur la santé mentale approuvée par l’Agence de la santé publique du Canada. Les patients âgés de 14 ans et plus qui se sont présentés à cinq services des urgences pour des raisons de santé mentale en 2020-2021 ont été inclus. L’issue principale, soit l’application de la loi P-38, a été obtenue à partir des dossiers médicaux. Des renseignements sur la trajectoire clinique et la prise en charge ont également été recueillis. Les risques relatifs (RR) ont été estimés à l’aide d’une régression de Poisson modifiée avec une variance robuste.

**Résultats:**

Parmi 15 021 visites aux services des urgences liées à la santé mentale, 860 (5,7% [IC à 95% : 5,4 à 6,1]) impliquaient l’application de la loi P-38. L’âge moyen était de 41,1 ± 16,5 ans, 46,1% des patients étant âgés de 25 à 44 ans et 54,4% étant des hommes. La plupart des patients vivaient de façon autonome (85,4%), sont arrivés par ambulance (80,5%) et avaient des antécédents documentés en matière de santé mentale (92,2%). Une proportion plus élevée de patients étaient démunis sur le plan économique (*p* = 0,020). Comparativement aux patients qui se sont rendus au service des urgences volontairement, les patients amenés contre leur gré en vertu de la loi P-38 ans étaient plus susceptibles de faire l’objet d’une contention (RR : 1,68 [IC À 95% : de 1,45 à 1,94]), d’être placés en confinement préventif (RR : 3,41 [IC à 95% : de 2,96 à 3,93]), de faire l’objet d’une évaluation multidisciplinaire (RR : 1,70 [IC À 95% : de 1,59 à 1,80]) et d’être hospitalisés (RR : 1,36 [IC À 95% : de 1,24 à 1,48].

**Conclusion:**

Près de 6% des visites au service des urgences liées à la santé mentale impliquaient l’application de la loi P-38, et touchaient de façon disproportionnée des personnes démunies sur le plan économique du début au milieu de l’âge adulte qui avaient des antécédents de troubles de santé mentale. Nos résultats suggèrent un recours à des mesures coercitives et à court terme, ce qui souligne la nécessité d’efforts stratégiques ciblés pour élargir les options d’intervention communautaire en cas de crise pour cette population vulnérable.

## Introduction

Mental health issues are increasingly prevalent across various countries.^1,2^ Even though mental health is considered a public health priority in Canada, accessing mental health care remains challenging.^1,3^ In fact, fewer than half of the population needing mental health services reported receiving effective, affordable, and quality care.^4^ As the number of individuals living with mental health conditions rises, there is a significant increase in acute events or crises.^1^ While this was initially highlighted in the early 2000s, it has worsened in the subsequent years and has been exacerbated since 2020 by the COVID-19 pandemic.^3^

Most jurisdictions have laws allowing professionals to have an individual assessed by a physician without their consent if they are considered to be at imminent risk of harming themselves or others.^5,6^ A key challenge is balancing safeguarding public health with upholding people's rights.^7^ In Canada, laws on involuntary commitment vary from province to province. While these laws have shown effectiveness in preventing violent acts and suicides, they also raise ethical and legal concerns regarding human rights. Canadian provincial mental health legislation shares a common architecture: statutory criteria, a mechanism for authorization, and procedural rights protections, but diverges on several clinically and legally significant dimensions.^8–10^ The central fault line opposes jurisdictions requiring evidence of imminent bodily danger to those that also permit intervention on the basis of substantial mental or physical deterioration, effectively enabling earlier preventive action.^8,10^ Ontario, Alberta, and British Columbia each permit such earlier intervention. By contrast, Québec's legislation does not include an explicit deterioration clause, placing it among the more restrictive provincial regimes with respect to the triggering threshold, a distinction with direct implications for how and when individuals reach the Emergency Department (ED).^8,10^

In the province of Quebec, the P-38.001 Act (thereafter P-38) was introduced in 1998, as the “Act respecting the protection of persons whose mental state presents a danger to themselves or others” under the provincial Minister of Health and Social Services (*Ministère de la Santé et des Services sociaux*).^6,11^ The provisions of this Act complement and bring modifications to both the Civil Code and the Code of Civil Procedure regarding confinement (i.e., preventive or temporary confinement) of individuals whose mental state poses a danger to themselves or others in a hospital or a health and social services institution, provided that the institution is equipped with the necessary facilities. At the request of a member of a crisis intervention unit or the person having parental authority, the Act allows a police officer or crisis worker, without requiring court authorization, to take an individual against their will to a designated institution or an ED.^6^ Contingent on the presence of more pressing medical emergencies, the person is examined by a physician or a specialized nurse practitioner who may place the person under preventive confinement (prior to or during a psychiatric examination) for up to 72 h. After this period, the person must be released, unless the court has ordered continued custody following a psychiatric evaluation. However, balancing the protection of patients’ rights, limited access to mental health care, and the challenges of stigmatization associated with involuntary commitment are significant issues.^12^ Recently, a report by the *Institut québécois de réforme du droit et de la justice* highlighted several significant concerns. For instance, the notion of immediate danger is fundamental to this law, but it is difficult to define and implement. Other challenges included: threats to rights and liberties from excessive restrictions, practical issues such as complex collaboration and resource shortages, barriers to justice such as time-intensive procedures, and a system marked by fragmentation, stigma, and fragile services.^11^

To promote transparency and gain insights into current practices, it is crucial to understand the characteristics of the individuals on whom the P-38 Act was enforced and how it affects patient care. Currently, limited data and evidence are available on the application of the Mental Health Act, especially in the province of Quebec. We aimed to determine the prevalence of P-38 use among ED patients with mental health presentations and to compare their characteristics, clinical course, and outcomes with those of patients who sought care voluntarily.

## Methods

### Study Design and Setting

This retrospective cohort study used data from the Public Health Agency of Canada-approved specialized mental health database (Canadian Hospitals Injury Reporting and Prevention Program-CHIRPP) operated at the CHU de Québec-Université Laval (a university-affiliated health centre comprising 5 teaching hospitals).^13^

The CHU de Québec-Université Laval comprises 5 teaching urban hospitals, 2 of which have dedicated psychiatric ED units. According to regional processes, all patients in the ED are initially assessed by an emergency physician before being referred to the psychiatric ED, if deemed necessary. In the psychiatric ED, a multidisciplinary team assesses the patient and determines whether hospitalization is required.

The CHU de Québec-Université Laval Research Ethics Board (#2024-7188) approved this project. Per the retrospective nature of this study, patient-informed written consent was waived. All data were denominalized prior to analyses. Our results are reported in accordance with the Strengthening the Reporting of Observational Studies in Epidemiology guidelines.^14^

### Population

Individuals aged 14 and older were included if they attended one of the participating EDs for a mental health issue between March 2020 and March 2021.

### Outcome Measures

Trained medical archivists abstracted sociodemographic and relevant clinical information from medical charts, including the presence of a previous mental health diagnosis. The reason for transport to the ED, as well as the physician's diagnostic impression or clinical manifestation of the mental health issues, were also collected. The reason for consultation is documented by triage nurses using the Canadian Triage and Acuity Scale.^15^ An emergency physician or a psychiatrist determined the diagnostic impression or clinical manifestation of the mental health issue. All diagnostic impressions and clinical manifestations were categorized to align as closely as possible with the *DSM-5* criteria.^16^ CHIRPP data is subjected to systematic quality control by the local program coordinator. No formal inter-rater reliability assessment was performed for this study, however, any discrepancies or uncertainties arising during abstraction are reviewed with the coordinator to ensure coding consistency.

Information regarding the socioeconomic status was unavailable in our database. Therefore, we used the Material and Social Deprivation Index from the *Institut National de Santé Publique du Québec* as a proxy.^17^ This 2-dimensional tool measures material and social deprivation in relatively homogeneous geographical units, known as dissemination areas.^17,18^ The index is derived from 6 socioeconomic indicators using a principal component analysis standardized by the Canadian population's age and sex (2021 Canadian Census). Dissemination areas are ranked from most privileged (Q1) to most deprived (Q5), with each quintile representing ∼20%. Since all 5 EDs are in the same region, we used the regional index.

This study's main outcome measure, the enforcement of the P-38 Act, was collected from the medical charts. We also collected information regarding the patient's care trajectory and management in the ED. This encompasses assessments by mental health professionals, the application of restraints (either physical or chemical), and the patient's disposition after leaving the ED.

### Statistical Analysis

Patients were divided into 2 groups: those for whom the P-38 Act was used, and those who “voluntarily” consulted in the ED. The term “voluntarily” refers strictly to the absence of P-38 enforcement and does not preclude other forms of social or clinical pressure that may have influenced the decision to seek care. Sociodemographic and clinical data are presented using frequencies for categorical variables and means (standard deviations) for continuous variables.

The Material and Social Deprivation Index linked patients’ postal codes, some of which were from excluded areas due to lower population density or the presence of institutions. The proportion of missing data is approximately 5%, and we excluded these cases from the analyses in accordance with current practice.

To compare the clinical course between the 2 groups, we applied a multivariable model with P-38 Act as the primary exposure alongside potential confounders (age, sex, residence, mode of arrival, hospital status and socio-economic status) for a set of outcomes (the use of restraints, preventive confinement, consultants, and hospitalization).

The categories of socio-economic status were obtained by combining the 10 material and social quantiles into the following 5 subsets: highly privileged material and social areas, average areas, materially privileged and socially deprived areas, highly privileged social and materially deprived areas, and highly deprived material and social areas.^17^

Relative risks (RRs) were calculated by using a modified Poisson regression model and 95% confidence intervals (CIs) were estimated with a robust variance estimator. All statistical analyses were performed using the Statistical Analysis System (SAS Institute, Cary, NC, USA, version 9.4), with statistical significance set at *P* < .05.

## Results

A total of 15,021 mental-health-related ED consultations (8,961 patients) were included in our analyses. As shown in Table 1, 860 consultations (547 unique patients) involved individuals subject to the P-38 Act, accounting for 5.7% (95% CI: 5.4-6.1%) of all consultations and 6.1% (95% CI: 5.6-6.6%) of all individuals. The mean age of the P-38 group was 41.1 ± 16.5 years, with 46.1% aged 25-44 years. They were most frequently male (54.4%), lived independently at home (85.4%), and arrived by ambulance (80.5%). Most (92.2%) had a documented history of mental health conditions. The most common prior mental health conditions in this group were mood (46.5%), personality (45.6%), and substance use disorders (40.8%). According to triage notes, the main reasons for consultation were suicidal ideation (48.3%) and behavioural problems (25.0%). Most of the P-38 group (60.3%) resided in socially disadvantaged dissemination areas (Q4: 27.5%, Q5: 32.8%). In contrast, material deprivation was more evenly distributed across quintiles (Q1: 18.8%, Q5: 25.1%).

**Table 1.** Sociodemographic Characteristics.

Characteristics	All Visits	P-38	Voluntary	*P*-Values
	*N* (%) 15,021 (100.0)	*N* (%) 860 (5.7)	*N* (%) 14,161 (94.3)	
**Number of unique patients among visits**	8961	547	8414	
**Number of visits per individual (min–max)**	1-44	1-18	1-44	
**Hospital**				<.001
Specialized in psychiatry/mental health	12,830 (85.4)	858 (99.8)	11,972 (84.5)	
Not specialized in psychiatry/mental health	2191 (14.6)	2 (0.2)	2189 (15.5)	
**Age**, mean ± standard deviation	40.7 ± 17.7	41.1 ± 16.5	40.7 ± 17.8	.496
14-24	2950 (19.6)	150 (17.4)	2800 (19.8)	.330
25-44	6652 (44.3)	396 (46.1)	6256 (44.2)	
45-64	3734 (24.9)	222 (25.8)	3512 (24.8)	
≥ 65	1685 (11.2)	92 (10.7)	1593 (11.3)	
**Sex, female**	6811 (45.3)	392 (45.6)	6419 (45.3)	.888
**Living arrangement**				<.001
Private home	11,951 (79.6)	734 (85.4)	11,217 (79.2)	
Senior housing, long-term care facility/supervised residence	1186 (7.9)	56 (6.5)	1130 (8.0)	
Shelter	1787 (11.9)	68 (7.9)	1719 (12.1)	
Other	97 (0.7)	2 (0.2)	95 (0.7)	
**Socio-economic status** ^&^				
Material deprivation				.020
Q1	2789 (18.6)	125 (14.5)	2664 (18.8)	
Q2	2004 (13.3)	110 (12.8)	1894 (13.4)	
Q3	2214 (14.7)	133 (15.6)	2081 (14.7)	
Q4	2820 (18.8)	187 (21.7)	2633 (18.6)	
Q5	3778 (25.2)	228 (26.5)	3550 (25.1)	
Social deprivation				.087
Q1	1266 (8.4)	84 (9.8)	1182 (8.4)	
Q2	1453 (9.7)	104 (12.1)	1349 (9.5)	
Q3	1864 (12.4)	109 (12.7)	1755 (12.4)	
Q4	4106 (27.3)	215 (25.0)	3891 (27.5)	
Q5	4916 (32.7)	271 (31.5)	4645 (32.8)	
**Means of arrival at ED**				
Ambulance	7617 (50.7)	692 (80.5)	6925 (48.9)	<.001
Other (includes prison van)	7404 (49.3)	168 (19.5)	7236 (51.1)	
**Accompanied^‡^**				
Police officers^¶¶^	2843 (18.9)	641 (74.5)	2202 (15.6)	<.001
Other (caregiver, intervener)	956 (6.4)	83 (9.7)	873 (6.2)	<.001
**Documented history of mental health disorders^‡^**				
Mood disorder(s)^#^	6020 (40.1)	400 (46.5)	5620 (39.7)	<.001
Neurocognitive disorder(s)	600 (4.0)	37 (4.3)	563 (4.0)	.591
Anxiety disorder(s)/phobia	4785 (31.9)	226 (26.3)	4559 (32.2)	<.001
Chronic alcoholism	4075 (27.1)	274 (31.9)	3801 (26.8)	.002
Personality disorder(s)^µ^	5353 (35.6)	392 (45.6)	4961 (35.0)	<.001
Suicide attempt(s)	3639 (24.2)	298 (34.7)	3341 (23.6)	<.001
No history of mental health disorder	1703 (11.3)	67 (7.8)	1636 (11.6)	<.001
Schizophrenia	1920 (12.8)	117 (13.6)	1803 (12.7)	.461
Psychosis	2447 (16.3)	187 (21.7)	2260 (16.0)	<.001
Suicidal ideation/dark thought(s)^†^	2139 (14.2)	175 (20.4)	1964 (13.9)	<.001
Other^¶^	4876 (32.5)	309 (35.9)	4567 (32.3)	.027
Substance use disorder and/or cannabis*	5137 (34.2)	351 (40.8)	4786 (33.8)	<.001
Other mental health disorder to be specified	779 (5.2)	44 (5.1)	735 (5.2)	1.000
Mental retardation (intellectual disability)	719 (4.8)	24 (2.8)	695 (4.9)	.004
Behavioral disorder	682 (4.5)	50 (5.8)	632 (4.5)	.076
**Reason for consultation (triage)^‡^**				
Behavioral problem	1891 (12.6)	215 (25.0)	1676 (11.8)	<.001
Suicidal ideation	4091 (27.2)	415 (48.3)	3676 (26.0)	<.001
Anxiety/situational crisis	2408 (16.0)	54 (6.3)	2354 (16.6)	<.001
Intoxication	3377 (22.5)	75 (8.7)	3302 (23.3)	<.001
Hallucinations	1091 (7.3)	69 (8.0)	1022 (7.2)	.379
Depressive mood	1182 (7.9)	14 (1.6)	1168 (8.3)	<.001
Other^‡‡^	985 (6.6)	51 (5.9)	934 (6.6)	.4789
Suicide attempt	775 (5.2)	56 (6.5)	719 (5.1)	.0678
Insomnia	376 (2.5)	2 (0.2)	374 (2.6)	<.001
Social problem	568 (3.8)	10 (1.2)	558 (3.9)	<.001

ED = Emergency Department.

‡ Categories are not mutually exclusive.

¶¶ According to triage notes. May have been affected by COVID restrictions.

# Adaptation, bipolar affective disorder, bipolar, depression, dysthymic disorder, affective dysregulation.

µ Borderline personality disorder, narcissistic, cluster B, antisocial, borderline, dependent, histrionic personality disorder, etc.

† Chronic suicidal risk, suicidal crisis.

¶ Aggression/violence, shock/post-traumatic stress, insomnia, potomania, social problems, eating disorder, obsessive-compulsive disorder, neurodevelopmental disorder, self-mutilation.

*Other than alcohol.

‡‡ Agitation/extreme violence, substance withdrawal, court order, self-mutilation.

& Patients living in shelters were excluded from this analysis, since it is based on postal codes.

Age and sex distributions were similar between the groups. Men accounted for the largest proportion, and more than half of the individuals were aged either 25-44 or ≥65 years. Most patients lived in their own homes (P-38: 85.4% vs. voluntary consultations: 79.2%, *P* < .001). We observed a significant difference in material deprivation (*P* = .020), with a higher proportion of patients considered deprived in the P-38 group (Q4 + Q5: 48.3% [P-38] vs. 43.7% [voluntary consultations]). There was no significant difference in social deprivation levels, although the highest proportion in both groups was in the 5th quintile (Q5). Most patients in the P-38 act group arrived by ambulance (80.5%), compared with only 48.9% in the voluntary group (*P* < .001). Supplemental Table S1 presents the characteristics of unique patients. During the study period, 413 individuals had one additional unplanned consultation, while 134 returned more than twice. The number of visits per individual, whether voluntary consultations or under P-38, ranged from 1 to 44 in the overall cohort and from 1 to 18 among those brought to the ED under the P-38 Act. Regarding the recurrent use of the P-38 Act, 700 individuals were subjected to it once, 52 twice, 10 three times, and 6 four times within the 13-month study period.

Several differences between groups in the documented history of mental health issues are presented in Table 1. Mood disorders (46.5% vs. 39.7%), personality disorders (45.6% vs. 35.0%), and substance use disorders (40.8% vs. 33.8%) were significantly more prevalent among patients brought under the P-38 Act (all *P* < .001). Suicidal ideation was the most common reason for consultation in both groups, but it was significantly more frequent in the P-38 group (48.3% vs. 26.0%, *P* < .001). The second most common reason for consultation in this group was behavioural issues (25.0%), whereas intoxication without suicidal intent was more common among patients who voluntarily sought care in the ED (23.3%).

Table 2 summarizes the clinical trajectory, mental health-related diagnostic impressions and manifestations, and post-ED disposition by group, while Supplemental Table S2 presents these findings stratified by age group. Most patients received multidisciplinary care involving ED physicians, psychiatrists and paramedical (i.e., social workers, nursing liaison) health-care professionals (P-38: 62.4%, voluntary consultations: 40.7%). Patients in the P-38 group were more frequently subjected to physical restraint (P-38: 16.5% vs. voluntary: 8.6%) and chemical restraint (P-38: 15.9% vs. voluntary consultations: 7.1%), and preventive confinement (27.2% vs. 6.6%). As shown in Supplemental Table S2, individuals aged 14 to 24 accounted for fewer consultations, restraints, and hospitalizations, but had higher rates of suicidal ideation compared with older age groups. This younger group also showed the lowest proportions of preventive confinement and hospital admission.

**Table 2.** Clinical Care, Diagnostic Impression/Clinical Manifestation and Disposition.

	All Visits *N* (%) 15,021	P-38 *N* (%) 860	Voluntary *N* (%) 14,161
**Consultants in the ED**			
No consultant	4,752 (31.6)	153 (17.8)	4,599 (32.5)
Paramedical	2,720 (18.1)	115 (13.4)	2,605 (18.4)
Psychiatry	1,436 (9.6)	55 (6.4)	1,381 (9.8)
Psychiatry and paramedical	6,113 (40.7)	537 (62.4)	5,576 (39.4)
**Restraints used^‡^**			
Physical (Argentino, pinel)	1,358 (9.0)	142 (16.5)	1,216 (8.6)
Chemical^#^	1,136 (7.6)	137 (15.9)	999 (7.1)
Isolation room	181 (1.2)	44 (5.1)	137 (1.0)
**Preventive confinement**	1,162 (7.7)	234 (27.2)	928 (6.6)
**Mental health-related diagnostic impression/clinical manifestation (ED physician/psychiatrist)^‡^**			
Suicidal ideation/dark thoughts^¶^	6,981 (46.5)	596 (69.3)	6,385 (45.1)
With plan	4,322 (28.8)	398 (46.3)	3,924 (27.7)
Suicide attempt(s)	926 (6.2)	65 (7.6)	861 (6.1)
Neurocognitive disorders and delirium	605 (4.0)	38 (4.4)	567 (4.0)
Mood disorders^†^	3,062 (20.4)	166 (19.3)	2,896 (20.5)
Anxiety disorders	2,170 (14.5)	23 (2.7)	2,147 (15.2)
Personality disorders	3,515 (23.4)	288 (33.5)	3,227 (22.8)
Substance use disorders	4,264 (28.4)	305 (35.5)	3,959 (28.0)
Psychotic disorders	1,775 (11.8)	165 (19.2)	1,609 (11.4)
Substance/medication-induced psychosis	854 (5.7)	76 (8.8)	778 (5.5)
Schizophrenia^&^	987 (6.6)	79 (9.2)	908 (6.4)
Behavioral disorders—agressivity	768 (5.1)	82 (9.5)	686 (4.8)
Other mental health disorder^µ^	1,316 (8.8)	78 (9.1)	1,238 (8.7)
Social problems*	680 (4.5)	42 (4.9)	638 (4.5)
Hallucination(s)	180 (1.2)	2 (0.2)	178 (1.3)
No mental health disorders^‡‡^	247 (1.6)	13 (1.5)	234 (1.7)
Obsessive-compulsive and related disorders	118 (0.8)	6 (0.7)	112 (0.8)
Nonsuicidal self-injury	266 (1.8)	19 (2.2)	247 (1.7)
**ED disposition**			
Discharged^##^	9,301 (61.9)	492 (57.2)	8,809 (62.2)
Hospital admission	4,415 (29.4)	351 (40.8)	4,064 (28.7)
Admission for mental health problems^¶¶^	3,545 (23.6)	315 (36.6)	3,230 (22.8)
Admission for other health reasons^††^	359 (2.4)	24 (2.8)	335 (2.4)
Admission for substance intoxication/withdrawal	465 (3.1)	10 (1.2)	455 (3.2)
Left without being seen or unauthorized departure	1,305 (8.7)	17 (2.0)	1288 (9.1)

ED = Emergency Department.

‡ Categories are not mutually exclusive.

# Haldol alone, Haldol-Ativan combination, Ketamine, Versed (Midazolam), etc.

¶ Suicidal threat/crisis, passive death ideation, suicidal ideation for secondary gain.

† Depressive disorders, bipolar and related disorders.

& Schizoaffective disorder, paranoid schizophrenia.

µ Neurodevelopmental disorder, feeding and eating disorder, known and stable psychiatric illness, Insomnia, post-traumatic shock, factitious disorder, hetero-aggressive ideation, somnolence, attachment disorder, homicidal ideation, gambling disorder, acute stress, gender dysphoria, nervous shock, somatization/somatoform disorder, etc.

*Homelessness, environmental exhaustion, poverty/social deprivation, etc.

‡‡ Crime/assault victim, loss of autonomy, court order without psychiatric decompensation.

## Includes sobering up in local resources.

¶¶ Regardless of specialty.

†† Other than intoxication/overdose/mental health problems.

Suicidal ideation was significantly more frequent in the P-38 group (69.3% vs. 45.1%). Substance-use (35.5% vs. 28.0%) and personality disorders (33.5% vs. 22.8%) were also more common diagnostic impressions in this group, whereas anxiety disorders were less frequent (2.7% vs. 15.2%). Patients under the P-38 Act were more often referred for psychiatric assessment. Regarding ED disposition, although most patients were discharged home, hospitalization was significantly more frequent among these patients (40.8% vs. 28.7%).

Table 3 presents the clinical course of patients after adjustment for potential confounders. Compared with voluntary ED patients, those brought under the P-38 Act were at higher risk of being physically restrained (RR: 1.68, 95% CI, 1.45-1.94) or placed under preventive confinement (RR: 3.41 [95% CI, 2.96-3.93]). They were also more likely to receive multidisciplinary care involving an ED physician, psychiatry, and paramedical professionals (RR: 1.70 [95% CI, 1.59-1.80]), and to be hospitalized (RR: 1.36 [95% CI, 1.24-1.48]).

**Table 3.** Adjusted^‡^ Relative Risk (RR) and 95% Confidence Intervals (CI) for main outcomes.

	Restraint Use*	Preventive Confinement	No Consultation	Paramedical Only	Psychiatry Only	Psychiatry and Paramedical	Consultation (other)	Hospital Admission
	*N* = 1724 RR [95% CI]	*N* = 1162 RR [95% CI]	*N* = 3891 RR [95% CI)	*N* = 2720 RR [95% CI]	*N* = 1436 RR [95% CI]	*N* = 6113 RR [95% CI]	*N* = 4752 RR [95% CI]	*N* = 4415 RR [95% CI]
**P38**								
Non-adjusted	2.22 [1.95-2.53]	4.15 [3.66-4.71]	1.00 [0.85-1.19]	0.73 [0.61-0.86]	0.66 [0.51-0.85]	1.59 [1.50-1.68]	0.55 [0.47-0.63]	1.42 [1.31-1.55]
Adjusted^‡^	1.68 [1.45-1.94]	3.41 [2.96-3.93]	0.87 [0.76-0.98]	0.80 [0.67-0.97]	0.68 [0.51-0.90]	1.70 [1.59-1.80]	0.63 [0.54-0.73]	1.36 [1.24-1.48]
**Age**								
14-24	1	1	1	1	1	1	1	1
25-44	1.67 [1.44-1.96]	1.69 [1.40-2.03]	0.61 [0.56-0.67]	0.93 [0.84-1.02]	0.90 [0.78-1.03]	1.00 [0.95-1.06]	1.05 [0.98-1.12]	1.45 [1.33-1.58]
45-64	1.03 [0.86-1.23]	1.59 [1.30-1.95]	0.55 [0.49-0.61]	0.95 [0.85-1.06]	0.96 [0.82-1.12]	1.09 [1.02-1.15]	0.92 [0.85-0.99]	1.63 [1.49-1.78]
≥ 65	1.02 [0.87-1.26]	1.22 [0.94-1.60]	0.42 [0.35-0.51]	1.12 [0.98-1.28]	1.17 [0.97-1.41]	0.99 [0.91-1.07]	0.80 [0.73-0.88]	2.11 [1.92-2.33]
**Sex**								
Female	1	1	1	1	1	1	1	1
Male	1.23 [1.12-1.36]	1.10 [0.98-1.24]	0.78 [0.72-0.84]	1.05 [0.98-1.13]	0.82 [0.73-0.91]	0.91 [0.88-0.96]	1.12 [1.07-1.17]	0.93 [0.88-0.98]
**Residence**								
Home	1	1	1	1	1	1	1	1
Senior housing	1.21 [1.01-1.45]	0.67 [0.50-0.87]	0.25 [0.18-0.36]	1.03 [0.89-1.19]	1.03 [0.84-1.28]	1.27 [1.18-1.37]	0.75 [0.66-0.85]	1.44 [1.33-1.57]
Shelter	1.67 [1.47-1.90]	1.14 [0.94-1.40]	0.26 [0.20-0.36]	1.06 [0.94-1.20]	0.62 [0.49-0.79]	0.74 [0.68-0.81]	1.05 [0.98-1.11]	0.82 [0.73-0.91]
**Mode of arrival**								
Other	1	1	1	1	1	1	1	1
Ambulance	3.25 [2.87-3.67]	1.25 [1.10-1.42]	0.65 [0.60-0.71]	0.87 [0.81-0.94]	0.80 [0.72-0.89]	0.77 [0.74-0.80]	1.58 [1.51-1.66]	0.84 [0.79-0.88]
**Hospital**								
Not specialized	1	1	1	1	—	—	1	1
Specialized in psychiatry	1.59 [1.36-1.87]	4.22 [3.04-5.86]	6.36 [4.76-8.50]	0.82 [0.74-0.90]	—	—	0.37 [0.35-0.38]	3.01 [2.63-3.43]
**Socio-economic status**								
Materially and socially privileged DA	1	1	1	1	1	1	1	1
DA with average material and social deprivation	0.90 [0.71-1.16]	1.00 [0.77-1.31]	1.11 [0.96-1.30]	0.90 [0.76-1.06]	1.00 [0.79-1.26]	1.02 [0.94-1.11]	1.01 [0.89-1.14]	0.95 [0.85-1.07]
Materially privileged but socially deprived DA	1.03 [0.84-1.28]	0.87 [0.68-1.11]	0.98 [0.85-1.13]	0.99 [0.86-1.15]	0.88 [0.71-1.26]	0.87 [0.80-0.94]	1.11 [0.99-1.24]	0.89 [0.77-0.99]
Socially privileged but materially deprived DA	0.91 [0.72-1.15]	1.02 [0.79-1.32]	1.08 [0.93-1.26]	0.97 [0.83-1.14]	1.00 [0.79-1.25]	1.02 [0.94-1.11]	0.96 [0.85-1.09]	1.00 [0.90-1.12]
Materially and socially deprived DA	1.14 [0.93-1.41]	1.04 [0.83-1.31]	0.86 [0.74-0.99]	0.96 [0.83-1.11]	1.03 [0.84-1.26]	0.86 [0.80-0.93]	1.14 [1.02-1.27]	0.95 [0.86-1.04]

DA = diffusion area; RR = relative risk; CI = confidence interval.

‡ Adjusted for all variables included in the table unless specified otherwise.

*Includes physical (Argentino, Pinel), chemical (Ativan, Haldol, Ketamine, Midazolam, etc.) and isolation room.

## Discussion

In our cohort of over 15,000 mental health–related ED consultations, approximately 1 in 20 involved enforcement of the P-38 Act. Patients in this group were most often male, in early to mid-adulthood, with a documented history of mental health conditions and a higher prevalence of material deprivation compared to those who sought care voluntarily. Despite receiving multidisciplinary management, patients under the P-38 Act experienced significantly higher rates of coercive measures and hospitalization, underscoring the clinical complexity of this population and the burden placed on emergency psychiatric services.

Consistent with prior work, we observed an overrepresentation of socially disadvantaged men in their mid-30s with substance use issues, a profile frequently associated with involuntary hospitalization.^19^ Our results also highlight a phenomenon of recurrence, as some P-38 patients returned to the ED multiple times during the study period, a pattern similarly documented in other settings, including Australia.^20^ This recurrence may reflect fragmented care and challenges in accessing post-ED resources for these individuals, who often require more than short-term crisis intervention. Combined with the overrepresentation of mood, personality, and substance use disorders, contrasting with the predominance of psychosis reported in international involuntary commitment cohorts,^19^ these findings suggest a distinct clinical profile among P-38 patients in Québec: one characterized by chronic, complex conditions rather than acute psychotic presentations. Consistent with this interpretation, 92.2% of P-38 patients had a documented history of mental health conditions, suggesting that most were already known to the mental health system, and material deprivation was significantly more prevalent in this group. Taken together, P-38 enforcement may disproportionately affect individuals with greater vulnerability, whose crises escalate to the point of imminent danger despite, or in the context of, prior contact with the mental health system.

Suicidal ideation emerged as the leading reason for consultation in both groups, based on triage and diagnostic information. However, behavioural issues were the second most frequent reason in the P-38 group, whereas intoxication without suicidal ideation predominated among those who voluntarily consulted in the ED. These findings suggest that P-38 enforcement was primarily applied to individuals posing a danger to themselves rather than to others.

Material deprivation in the absence of social deprivation was not associated with P-38 enforcement or related outcomes. However, recent literature indicates that material deprivation affects mental health, notably through its interaction with social deprivation.^21^ In addition, a nationwide longitudinal study of Canadian adults found increased psychological distress in socially deprived neighbourhoods.^22^ In our study, the largest proportion of patients in both groups fell within the 5th quintile of social deprivation. This suggests that social deprivation, characterized by social isolation, lower educational attainment, and weaker community cohesion, may contribute to vulnerability to mental health problems and greater reliance on hospital services. Patients under the P-38 Act did not differ from those who voluntarily presented to the ED in this regard. Nevertheless, these findings underscore the importance of reinforcing social ties, promoting community participation, and strengthening social capital as potential protective factors.

Within Canada, provincial comparisons are particularly instructive. Ontario's strong protection of a capable patient's right to refuse treatment has been associated with prolonged detention without clinical improvement, whereas British Columbia's closer link between involuntary admission and imposed treatment reflects a more paternalistic approach.^8,10^ Alberta, having reformed its legislation to permit earlier intervention based on deterioration rather than imminent danger, has developed community treatment orders as alternatives to inpatient admission.^8,23^ Québec's danger-threshold model, combined with its reliance on the ED as the primary entry point for P-38 apprehensions, may contribute to the comparatively higher rates of coercive measures observed in our study.

Regarding the clinical trajectory, our results suggest that the application of the P-38 Act may have facilitated referrals to psychiatric assessment and multidisciplinary care in a larger proportion of P-38 cases. This contrasts with international studies showing that involuntary hospitalization does not automatically lead to improved access to specialized mental health care, with substantial cross-national variability reported in both admission practices and the involvement of psychiatrists and multidisciplinary teams.^19,24^

Rates of physical restraint and preventive confinement in our cohort exceeded those reported in comparable international settings, including Australia and across a recent international scoping review.^25,26^ Our higher rates of psychiatric admission may suggest a lower threshold for hospitalization in Quebec, greater clinical severity, suboptimal triage, or challenging access to ambulatory resources for follow-up and community stabilization.

In 2023, Quebec's Health Ministry mandated the *Institut québécois de réforme du droit et de la justice* to conduct a review of P-38, underscoring the lack of recent data on its application and raising a striking observation: given the exceptional nature of P-38, how is it used more than 16,000 times annually?^11^ Our study provides complementary evidence on this question, documenting prevalence, sociodemographic characteristics, and clinical pathways that are likely relevant to both clinicians and policy decision-makers. Notably, the primary intent of P-38 is security rather than treatment, as the legal focus is exclusively on imminent danger, with involuntary treatment requiring a separate Superior Court order under the Civil Code. This legal framework likely shapes the clinical course of patients brought to the ED through this mechanism and helps explain the comparatively high rates of coercive measures and subsequent discharge home observed in our cohort.

Under these conditions, it is unsurprising that most of our P-38 patients were discharged home. Whether the application of P-38 is overly frequent remains uncertain, but this should not overshadow the need for effective crisis intervention strategies. When individuals cannot safely remain alone, expanding access to on-site or community-based mental health resources may be preferable to defaulting to the ED. In our region, however, EDs remain the sole entry point for patients brought under the P-38 Act. Many likely stabilize during the waiting period before physician assessment, which may explain the high proportion of discharges directly from the ED. Confidentiality rules prevent ED physicians from sharing consultation information with community services, resulting in parallel systems that operate in silos. This fragmentation, combined with limited resources and deficient communication, further compounds the challenges associated with continuity of care for this population.

This study has limitations inherent to its retrospective design. Reliance on medical notes introduces the possibility of missing or incomplete data, which may have led to selection bias when identifying patients with mental health issues or information bias if P-38 enforcement was inaccurately recorded. Nevertheless, data were extracted by trained medical archivists and underwent thorough quality validation by the local program coordinator. Moreover, given the medico-legal obligations surrounding P-38 enforcement, systematic underreporting by physicians or triage nurses is highly unlikely. The brief length of stay and rapid evaluations in the ED also limit the ability to establish definitive mental health diagnoses, many of which require a comprehensive psychiatric assessment. As a result, diagnostic impressions may not always reflect precise diagnoses. To mitigate this, each file was carefully reviewed, and impressions were reclassified as closely as possible according to DSM-5 categories.^16^ With respect to statistical analyses, residual confounding cannot be entirely excluded, although most covariates were well-defined and consistently measured. Finally, while some strata had smaller sample sizes resulting in wider confidence intervals, most estimates were accompanied by relatively narrow intervals, which supports the precision of our findings. Although bed availability may influence disposition decisions at the margins, the legal framework of P-38 requires that patients be retained if deemed to pose an ongoing danger. This suggests that discharge-home decisions primarily reflect clinical judgment rather than capacity constraints alone. Nevertheless, bed availability was not captured in our dataset and cannot be entirely excluded as a contextual factor, particularly for patients near the clinical threshold for admission.

## Conclusion

Enforcement of the P-38 Act accounted for nearly 6% of mental health–related ED visits, representing the first comprehensive description of this population in a Québec emergency setting. Patients were disproportionately from materially deprived areas and those in early to mid-adulthood, with a prior history of mental health disorders, suggesting that P-38 enforcement may capture individuals whose crises escalate in the context of inadequate access to upstream care. Our findings suggest a substantial reliance on coercive and short-term measures in Quebec, underscoring the need for targeted policy efforts to expand community-based crisis intervention alternatives and improve timely access to mental health care for this vulnerable population.

## Supplemental Material

sj-docx-1-cpa-10.1177_07067437261468893 - Supplemental material for One in Twenty: Prevalence and Outcomes of Patients Brought to EDs Under Québec's P-38 Mental Health Act—A Retrospective Cohort Study: Un patient sur vingt : prévalence et résultats chez les patients ayant été admis au service des urgences en vertu de la loi p-38 du Québec sur la santé mentale—Étude de cohorte rétrospective Supplemental material, sj-docx-1-cpa-10.1177_07067437261468893 for One in Twenty: Prevalence and Outcomes of Patients Brought to EDs Under Québec's P-38 Mental Health Act—A Retrospective Cohort Study: Un patient sur vingt : prévalence et résultats chez les patients ayant été admis au service des urgences en vertu de la loi p-38 du Québec sur la santé mentale—Étude de cohorte rétrospective by Marie-Soleil Dumont, Valérie Boucher, Pier-Alexandre Tardif, Ann-Pier Gagnon, Axel Benhamed, Pierre-Gilles Blanchard, Audrey-Anne Dumais-Michaud, Marcel Émond and Eric Mercier in The Canadian Journal of Psychiatry

sj-docx-2-cpa-10.1177_07067437261468893 - Supplemental material for One in Twenty: Prevalence and Outcomes of Patients Brought to EDs Under Québec's P-38 Mental Health Act—A Retrospective Cohort Study: Un patient sur vingt : prévalence et résultats chez les patients ayant été admis au service des urgences en vertu de la loi p-38 du Québec sur la santé mentale—Étude de cohorte rétrospective Supplemental material, sj-docx-2-cpa-10.1177_07067437261468893 for One in Twenty: Prevalence and Outcomes of Patients Brought to EDs Under Québec's P-38 Mental Health Act—A Retrospective Cohort Study: Un patient sur vingt : prévalence et résultats chez les patients ayant été admis au service des urgences en vertu de la loi p-38 du Québec sur la santé mentale—Étude de cohorte rétrospective by Marie-Soleil Dumont, Valérie Boucher, Pier-Alexandre Tardif, Ann-Pier Gagnon, Axel Benhamed, Pierre-Gilles Blanchard, Audrey-Anne Dumais-Michaud, Marcel Émond and Eric Mercier in The Canadian Journal of Psychiatry

sj-docx-3-cpa-10.1177_07067437261468893 - Supplemental material for One in Twenty: Prevalence and Outcomes of Patients Brought to EDs Under Québec's P-38 Mental Health Act—A Retrospective Cohort Study: Un patient sur vingt : prévalence et résultats chez les patients ayant été admis au service des urgences en vertu de la loi p-38 du Québec sur la santé mentale—Étude de cohorte rétrospective Supplemental material, sj-docx-3-cpa-10.1177_07067437261468893 for One in Twenty: Prevalence and Outcomes of Patients Brought to EDs Under Québec's P-38 Mental Health Act—A Retrospective Cohort Study: Un patient sur vingt : prévalence et résultats chez les patients ayant été admis au service des urgences en vertu de la loi p-38 du Québec sur la santé mentale—Étude de cohorte rétrospective by Marie-Soleil Dumont, Valérie Boucher, Pier-Alexandre Tardif, Ann-Pier Gagnon, Axel Benhamed, Pierre-Gilles Blanchard, Audrey-Anne Dumais-Michaud, Marcel Émond and Eric Mercier in The Canadian Journal of Psychiatry
