# Supplementary material for: One in Twenty: Prevalence and Outcomes of Patients Brought to EDs Under Québec's P-38 Mental Health Act—A Retrospective Cohort Study: Un patient sur vingt : prévalence et résultats chez les patients ayant été admis au service des urgences en vertu de la loi p-38 du Québec sur la santé mentale—Étude de cohorte rétrospective
Source: Can J Psychiatry. 2026 Jul 27:07067437261468893. Online ahead of print. doi: 10.1177/07067437261468893 (PMC13407664; doi:10.1177/07067437261468893)
Supplement: sj-docx-1-cpa-10.1177_07067437261468893 - Supplemental material for One in Twenty: Prevalence and Outcomes of Patients Brought to EDs Under Québec's P-38 Mental Health Act—A Retrospective Cohort Study: Un patient sur vingt : prévalence et résultats chez les patients ayant été admis au service des ur [file sj-docx-1-cpa-10.1177_07067437261468893.docx]

**Supplementary Table 1. Sociodemographic characteristics according to the number of visits – Unique patients**

| **Characteristics** | All patients (1^st^ visit) | 1 visit (no P-38) | ≥ 2 visits (no P-38) | P38 with 1 visit* | P38 with ≥ 2 visits* |
| --- | --- | --- | --- | --- | --- |
|  | N (%)  8961 (100.0) | N (%)  6150 (68.6) | N (%)  2264 (25.3) | N (%)  413 (4.6) | N (%)  134 (1.5) |
| **Hospital** |  |  |  |  |  |
| Specialized in psychiatry/mental health | 7849 (87.6) | 5191 (84.5) | 2109 (93.2) | 411 (99.5) | 134 (100.0) |
| Not specialized in psychiatry/mental health | 1113 (12.4) | 956 (15.5) | 155 (6.8) | 2 (0.5) | 0 (0.0) |
| **Age**, mean ± standard deviation | 40.9 (19.1) | 40.8 (20.0) | 40.9 (17.2) | 41.9 (17.9) | 42.0 (15.2) |
| 14-24 | 2030 (2.7) | 1524 (24.8) | 412 (18.2) | 77 (18.6) | 17 (12.7) |
| 25-44 | 3674 (41.0) | 2408 (39.2) | 1019 (45.0) | 180 (43.6) | 67 (50.0) |
| 45-64 | 2043 (22.8) | 1312 (21.3) | 595 (26.3) | 101 (24.5) | 35 (26.1) |
| ≥ 65 | 1215 (13.6) | 906 (14.7) | 238 (10.5) | 55 (13.2) | 15 (11.2) |
| **Sex, female** | 4350 (48.5) | 3093 (50.3) | 1015 (44.8) | 186 (45.0) | 55 (41.0) |
| **Living arrangement** |  |  |  |  |  |
| Private home/apartment/condo | 7692 (85.8) | 5411 (88.0) | 1796 (79.3) | 370 (89.6) | 115 (85.8) |
| Senior housing, long-term care facility/supervised residence | 741 (8.3) | 496 (8.1) | 207 (9.1) | 27 (6.5) | 11 (8.2) |
| Shelter | 468 (5.2) | 198 (3.2) | 245 (10.8) | 16 (3.9) | 8 (6.0) |
| Other | 61 (0.7) | 45 (0.7) | 16 (0.7) | 0 (0.0) | 0 (0.0) |
| **Socio-economic status**^&^ |  |  |  |  |  |
| Material deprivation |  |  |  |  |  |
| Q1 | 1427 (15.9) | 978 (1.7) | 379 (18.1) | 52 (13.7) | 18 (14.8) |
| Q2 | 1338 (14.9) | 971 (17.1) | 297 (14.1) | 53 (14.0) | 17 (13.9) |
| Q3 | 1426 (15.9) | 987 (17.3) | 346 (16.5) | 73 (19.2) | 20 (16.4) |
| Q4 | 1740 (19.4) | 1196 (21.0) | 422 (20.1) | 100 (26.3) | 22 (18.0) |
| Q5 | 2188 (24.2) | 1464 (25.7) | 594 (28.3) | 89 (23.4) | 41 (33.6) |
| Social deprivation |  |  |  |  |  |
| Q1 | 933 (10.4) | 677 (11.9) | 200 (9.5) | 45 (11.8) | 11 (9.0) |
| Q2 | 995 (11.1) | 735 (12.9) | 197 (9.4) | 56 (14.7) | 7 (5.7) |
| Q3 | 1287 (14.4) | 942 (16.5) | 270 (12.9) | 58 (15.3) | 17 (13.9) |
| Q4 | 2106 (23.5) | 1400 (24.6) | 584 (27.8) | 87 (22.9) | 35 (28.7) |
| Q5 | 2798 (31.2) | 1842 (32.3) | 787 (37.5) | 121 (31.8) | 48 (39.3) |
| **Means of arrival at ED** |  |  |  |  |  |
| Ambulance | 4295 (47.9) | 2721 (44.2) | 1121 (49.5) | 340 (82.3) | 113 (84.3) |
| Other (includes prison van) | 4666 (52.1) | 3429 (55.8) | 1143 (50.5) | 73 (17.7) | 21 (15.7) |
| **Accompanied^‡^** |  |  |  |  |  |
| Police^¶¶^ | 1609 (18.0) | 776 (12.6) | 430 (19.0) | 303 (73.4) | 99 (73.9) |
| Other (caregiver, intervener) | 561 (6.3) | 386 (6.3) | 125 (5.5) | 39 (9.4) | 11 (8.2) |
| **Documented history of mental health disorders^‡^** |  |  |  |  |  |
| Mood disorder(s)^#^ | 3381 (37.7) | 2199 (35.8) | 949 (41.9) | 167 (40.4) | 66 (49.3) |
| Neurocognitive disorder(s) | 441 (4.9) | 342 (5.6) | 70 (3.1) | 25 (6.0) | 4 (3.0) |
| Anxiety disorder(s)/phobia | 2658 (29.7) | 1785 (29.0) | 738 (32.6) | 99 (24.0) | 36 (27.0) |
| Chronic alcoholism | 1782 (19.9) | 998 (16.2) | 642 (28.3) | 87 (21.1) | 55 (41.0) |
| Personality disorder(s)^µ^ | 2191 (24.5) | 1155 (18.8) | 844 (37.3) | 124 (30.0) | 68 (50.8) |
| Suicide attempt(s) | 1708 (19.1) | 1011 (16.4) | 534 (23.6) | 110 (26.6) | 53 (39.6) |
| No history of mental health disorder | 1528 (17.1) | 1291 (21.0) | 174 (7.7) | 55 (13.3) | 7 (5.2) |
| Schizophrenia | 868 (9.7) | 449 (7.3) | 360 (15.9) | 35 (8.5) | 24 (17.9) |
| Psychosis | 1011 (11.3) | 558 (9.1) | 360 (15.9) | 61 (14.8) | 32 (23.9) |
| Suicidal ideation/dark thought(s)^†^ | 906 (10.1) | 552 (9.0) | 267 (11.8) | 66 (16.0) | 21 (15.7) |
| Other^¶^ | 2587 (28.9) | 1695 (27.6) | 717 (31.7) | 124 (30.0) | 51 (38.1) |
| Substance use disorder and/or cannabis^*^ | 2286 (25.5) | 1276 (20.8) | 818 (36.1) | 127 (30.8) | 65 (48.5) |
| Other mental health disorder to be specified | 350 (3.9) | 200 (3.3) | 126 (5.6) | 18 (4.4) | 6 (4.5) |
| Mental retardation (intellectual disability) | 263 (2.9) | 154 (2.5) | 98 (4.3) | 4 (0.8) | 7 (5.2) |
| Behavioral disorder | 303 (3.4) | 184 (3.0) | 89 (3.9) | 23 (5.6) | 7 (5.2) |
| **Reason for consultation (triage)^‡^** |  |  |  |  |  |
| Behavioral problem | 1108 (12.4) | 635 (10.3) | 332 (14.7) | 102 (24.7) | 39 (29.1) |
| Suicidal ideation | 2441 (27.2) | 1591 (25.9) | 589 (26.0) | 202 (48.9) | 59 (44.0) |
| Anxiety/situational crisis | 1628 (18.2) | 1209 (19.7) | 382 (16.9) | 24 (5.8) | 13 (9.7) |
| Intoxication | 1733 (19.3) | 1231 (20.0) | 458 (20.2) | 31 (7.5) | 13 (9.7) |
| Hallucinations | 612 (6.8) | 394 (6.4) | 178 (7.9) | 29 (7.0) | 11 (8.2) |
| Depressive mood | 849 (9.5) | 649 (10.6) | 190 (8.4) | 8 (1.9) | 2 (1.5) |
| Other^‡‡^ | 520 (5.8) | 321 (5.2) | 169 (7.5) | 21 (0.1) | 8 (6.0) |
| Suicide attempt | 483 (5.4) | 328 (5.3) | 116 (5.1) | 34 (8.2) | 5 (3.7) |
| Insomnia | 255 (2.9) | 191 (3.1) | 63 (2.8) | 0 (0.0) | 1 (0.8) |
| Social problem | 276 (3.1) | 184 (3.0) | 86 (3.8) | 5 (1.2) | 1 (0.8) |

ED: Emergency Department;

* Other than alcohol;

^‡^ Categories are not mutually exclusive;

^&^ Patients living in shelters were excluded from this analysis, since it is based on postal codes;

^¶¶^ According to triage notes. May have been affected by COVID restrictions;

^#^ Adaptation, bipolar affective disorder, bipolar, depression, dysthymic disorder, affective dysregulation;

^µ^ Borderline personality disorder, narcissistic, cluster B, antisocial, borderline, dependent, histrionic personality disorder, etc;

^†^ Chronic suicidal risk, suicidal crisis;

^¶^ Aggression/violence, shock/post-traumatic stress, insomnia, potomania, social problems, eating disorder, obsessive-compulsive disorder, neurodevelopmental disorder, self-mutilation;

^‡‡^ Agitation/extreme violence, substance withdrawal, court order, self-mutilation.
