# Supplementary material for: One in Twenty: Prevalence and Outcomes of Patients Brought to EDs Under Québec's P-38 Mental Health Act—A Retrospective Cohort Study: Un patient sur vingt : prévalence et résultats chez les patients ayant été admis au service des urgences en vertu de la loi p-38 du Québec sur la santé mentale—Étude de cohorte rétrospective
Source: Can J Psychiatry. 2026 Jul 27:07067437261468893. Online ahead of print. doi: 10.1177/07067437261468893 (PMC13407664; doi:10.1177/07067437261468893)
Supplement: sj-docx-2-cpa-10.1177_07067437261468893 - Supplemental material for One in Twenty: Prevalence and Outcomes of Patients Brought to EDs Under Québec's P-38 Mental Health Act—A Retrospective Cohort Study: Un patient sur vingt : prévalence et résultats chez les patients ayant été admis au service des ur [file sj-docx-2-cpa-10.1177_07067437261468893.docx]

**Supplementary Table 2. Clinical care, diagnostic impression/clinical manifestation and disposition according to age groups**

|  | **Aged 14-24** | | **Aged 25-44** | | **Aged 45-64** | | **Aged ≥ 65** | |
| --- | --- | --- | --- | --- | --- | --- | --- | --- |
|  | **All visits**  **N = 2800** | **P38**  **N = 150** | **All visits**  **N = 6256** | **P38**  **N = 396** | **All visits**  **N = 3512** | **P38**  **N = 222** | **All visits**  **N = 1593** | **P38**  **N = 92** |
| **Consultants** |  |  |  |  |  |  |  |  |
| No consultant | 754 (26.9) | 18 (12.0) | 811 (13.0) | 71 (17.9) | 386 (11.0) | 53 (23.9) | 127 (8.0) | 11 (12.0) |
| Paramedical | 1681 (60.0) | 26 (17.3) | 3452 (55.2) | 46 (11.6) | 2082 (59.3) | 26 (11.7) | 966 (60.6) | 17 (18.5) |
| Psychiatry | 1467 (52.4) | 12 (8.0) | 2895 (46.3) | 22 (5.6) | 1780 (50.7) | 13 (5.9) | 815 (51.2) | 8 (8.7) |
| Psychiatry and paramedical | 1161 (41.5) | 94 (62.7) | 2346 (37.5) | 257 (64.9) | 1444 (41.1) | 130 (58.6) | 625 (39.2) | 56 (60.9) |
| **Restraint use** |  |  |  |  |  |  |  |  |
| Physical | 156 (5.6) | 21 (14.0) | 709 (11.3) | 74 (18.7) | 238 (6.8) | 33 (14.9) | 113 (7.1) | 14 (15.2) |
| Chemical | 125 (4.5) | 20 (13.3) | 586 (9.4) | 75 (18.9) | 209 (6.0) | 29 (13.1) | 80 (5.0) | 13 (14.1) |
| Isolation room | 33 (4.4) | 9 (6.0) | 72 (1.2) | 22 (5.6) | 20 (0.6) | 10 (4.5) | 12 (0.8) | 3 (3.3) |
| **Preventive confinement** | 124 (4.4) | 39 (26.0) | 480 (7.7) | 112 (28.3) | 256 (7.3) | 59 (26.6) | 68 (4.3) | 24 (26.1) |
| **Hospital admission** | 527 (18.8) | 50 (33.3) | 1407 (22.5) | 157 (39.7) | 881 (25.1) | 80 (36.0) | 415 (26.0) | 28 (30.4) |
| **Mental health-related diagnostic impression/clinical manifestation (ED physician/psychiatrist)^‡^** | | | | | | | | |
| Neurocognitive disorders | 6 (0.2) | 0 (0.0) | 15 (0.2) | 0 (0.0) | 48 (1.4) | 1 (0.5) | 498 (31.2) | 37 (40.2) |
| Mood disorders | 671 (23.9) | 30 (20.0) | 1086 (17.4) | 66 (16.7) | 735 (20.9) | 44 (19.8) | 404 (25.3) | 26 (28.3) |
| Anxiety disorders | 641 (22.9) | 6 (4.0) | 802 (12.8) | 7 (1.8) | 436 (12.4) | 7 (3.2) | 268 (16.8) | 3 (3.3) |
| Personality disorders | 732 (26.1) | 59 (39.3) | 1480 (23.7) | 143 (36.1) | 798 (22.7) | 67 (30.2) | 217 (13.6) | 19 (20.7) |
| Schizophrenia* | 236 (8.4) | 26 (17.3) | 1220 (19.5) | 122 (30.8) | 673 (19.2) | 60 (27.0) | 306 (19.2) | 17 (18.5) |
| Substance use disorder | 547 (19.5) | 48 (32.0) | 2160 (34.5) | 161 (40.7) | 1077 (30.7) | 79 (35.6) | 175 (11.0) | 17 (18.5) |
| Suicidal ideation | 1530 (54.6) | 124 (82.7) | 2509 (40.1) | 260 (65.7) | 1688 (48.1) | 160 (72.1) | 658 (41.3) | 52 (56.2) |

‡ Categories are not mutually exclusive

*Includes substances-induced psychosis and hallucinations
